# Long transmaxillary implants improve oral health-related quality of life of patients with atrophic jaws-a case series

**DOI:** 10.1186/s40729-021-00300-7

**Published:** 2021-03-15

**Authors:** Gino Kopp, João Cezar Zielak, Suyany Gabriely Weiss, Fernanda Kopp, Tatiana Miranda Deliberador

**Affiliations:** 1 Latin American Institute of Dental Research and Education, ILAPEO, R. Jacarezinho, 656, Curitiba, PR 80710-150 Brazil; 2 Kopp Institute, Curitiba, PR Brazil

**Keywords:** Quality of life, Dental implants, Zygoma, Jaw, Edentulous

## Abstract

**Background:**

The advancement of contemporary dentistry is related to the improvement of existing techniques, materials, and technology, consistently for improving people’s oral health, which can ultimately reflect better quality of life. This study aimed to evaluate the oral-health-related quality of life (OHRQoL) of patients with atrophic jaws, who reported for the placement of long transmaxillary implants and posterior prosthetic rehabilitation. Twelve patients (*n* = 12), of both sexes, with a mean age of 55.83 ± 2.78 years, who were unable to receive conventional implants immediately because of lack of bone, received two long transmaxillary implants in a horizontal position, anteroposteriorly, one on each side, from the canine pillar to the maxillary tuberosity. After 6 months, the conventional clinical sequence for fabricating a fixed prosthesis type protocol or removable prosthesis type overdenture (MK1® system) was performed, when required to recover the lip volume. The *Oral Health Impact Profile* questionnaire (OHIP-14) was applied preoperatively and 6 months after rehabilitation using a prosthesis on the implants. The results were statistically analyzed using a significance level of 0.05.

**Results:**

An improvement in the perception of OHRQoL was observed between the pre- and postoperative periods in the OHIP-14 total score and the domains related to functional limitation, physical pain, psychological discomfort, psychological disability, social disability, and handicap (*p* < 0.05).

**Conclusion:**

It may be concluded that transmaxillary implant rehabilitation improves the *OHRQoL*.

## Background

The introduction of osseointegrated implants is considered one of the greatest breakthroughs in dentistry [4]. Dental implants have been used in edentulous jaws to improve the retention and stability of complete dentures. Attachment to implants, in addition to improving stability and functional aspects, increases patient satisfaction [7, 8]. In addition, implant connection improves neuromuscular activity, thereby improving masticatory function in edentulous patients [17]. Additionally, it has been demonstrated that individuals with implants may achieve double of the masticatory forces compared with those with conventional dentures [18].

Several factors should be considered for the rehabilitation of edentulous patients, particularly those with the severely resorbed or resected maxilla, such as the bone quality and quantity, as well as the condition of the soft tissues [11]. Zygomatic implants have been shown to provide a stable and predictable alternative for the rehabilitation of patients with severe maxillary bone loss [13]. Patient-related factors such as mental and oral health, esthetic demands, and treatment perceptions should be equally considered [15]. Tooth loss may impair individuals’ lives, particularly causing psychological effects. These include lack of confidence and self-esteem, avoiding laughing in public, or even socializing in general [15].

The term “oral-health-related quality of life” is now extensively used and accepted worldwide [12]. The influence of dentures and dental implants on the quality of life (OHRQoL) related to the oral health of the patient can guide the clinician to provide the best treatment plan. Thus, the Oral Health Impact Profile (OHIP)-14 questionnaire allows the translation of patients’ perceptions and expectations [1].

Considering that dental implants may improve OHRQoL, this retrospective case series aimed to evaluate the OHRQoL of patients with atrophic jaws, who reported for placement of long transmaxillary implants and prosthetic rehabilitation.

## Materials and methods

### Ethical aspects

This retrospective case series was approved by the local Research Ethics Committee (CAAE 98088718.5.0000.0093). Patients were informed of the nature of the study and procedures, according to the Free and Informed Consent Term. Additionally, this study followed the Helsinki guidelines [19].

### Study design

Twelve adult patients of both sexes were evaluated. They were users of total prostheses on totally edentulous maxillae with severe resorption, which prevented the placement of conventional implants between the canine and maxillary tuberosity.

These patients had good health conditions and psychological disposition to undergo common oral surgery procedures under local anesthesia, in addition to the clinical procedures necessary for the fabrication of prostheses. Such patients underwent (1) preoperative examination for planning, (2) transmaxillary implant placement with or without using the homologous bone (used for grafting in the maxillary sinus, simultaneously with the implant placement, when necessary), and (3) prosthetic rehabilitation after the period of osseointegration of the implants (6 months).

Patients, who demonstrated the presence of systemic pathologies (hematological, diabetes, autoimmune diseases), infections, or apparent inflammation in the oral cavity, maxillary sinuses, or any region of the stomatognathic system, were excluded. Patients, who used bisphosphonates and who underwent the head and neck irradiation in a period of 60 months before this study, were also eliminated. Smokers, patients with bruxism or severe jaw tightening, those with poor oral hygiene, and those who were unable to assume the costs of homologous bone or prosthetic rehabilitation after implant osseointegration were also excluded.

**Fig. 1:**
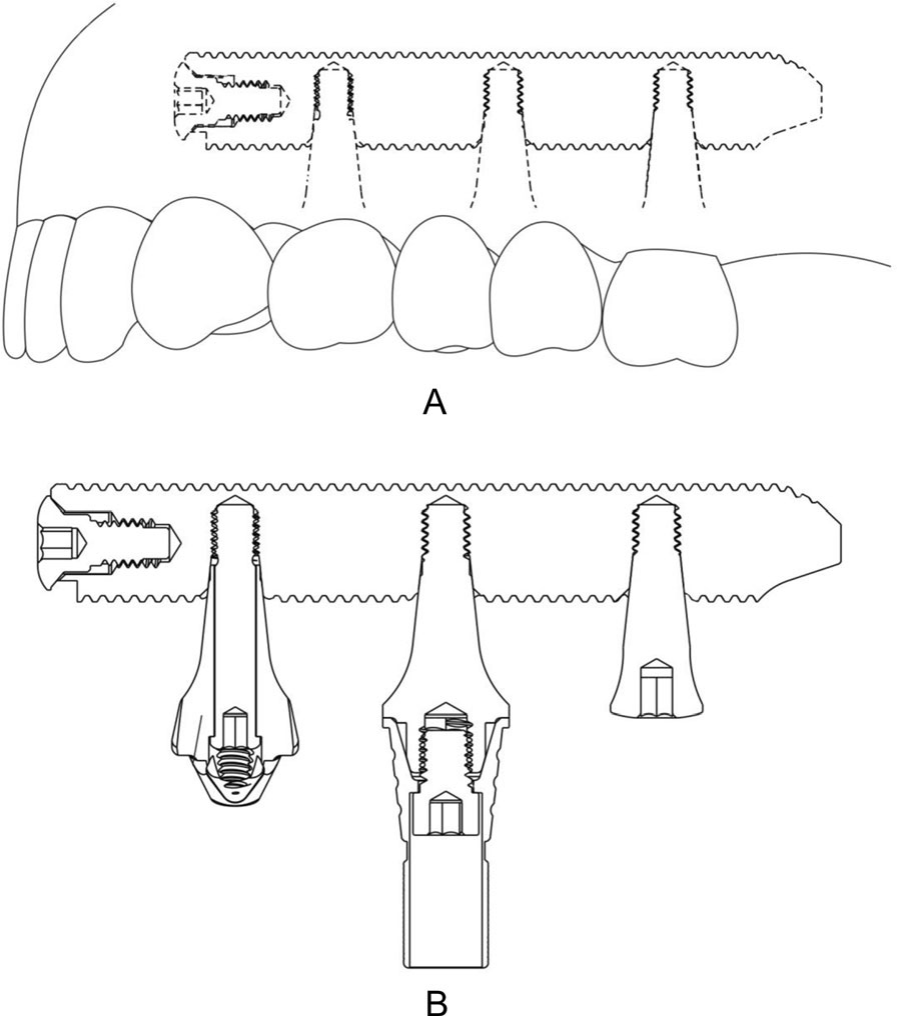
Schematic drawing of the transmaxillary implant. Perforation is made using the 2.0 mm lance implant, for giving guidance and stability to the next drill, using the 3.0 mm implant

The procedures were performed during a period of 5 years (considering the date of the first appointment and the evaluation of the last image). Surgical guides and bio-models (three-dimensional printing) were prepared for the placement of transmaxillary implants (Kopp, Curitiba, Paraná, Brazil) horizontally (Fig. 1). The patients underwent transmaxillary implant placement and prosthesis delivery at the clinic of Instituto Kopp, Curitiba, Paraná, Brazil. The prostheses received immediate loading or late loading, depending on local and surgical conditions. In the pre- and postoperative evaluations, all patients were assessed for OHRQoL using the OHIP-14 questionnaire.

The transmaxillary implant is more conservative than the zygomatic implant, because it is installed only in the lower third of the maxilla. The zygomatic implant can reach noble regions of the face and can cause serious risks of injury to support structures, including the face, eye socket, and infratemporal fossa. Long transmaxillary implants are positioned horizontally in the maxillary bone, unlike the zygomatic implants installed in a vertical position. They are 4.3-mm-thick cylindrical implants that vary in length from 23 to 43 mm and have niches in the Morse cone platform next to the body, to couple the prosthetic components for future prosthetic rehabilitation. These transmaxillary implants are anchored from the canine abutment to the maxillary tuberosity for stability and may or may not proceed with the sinus membrane elevation technique for installation.

#### Preoperative planning

**Fig. 2:**
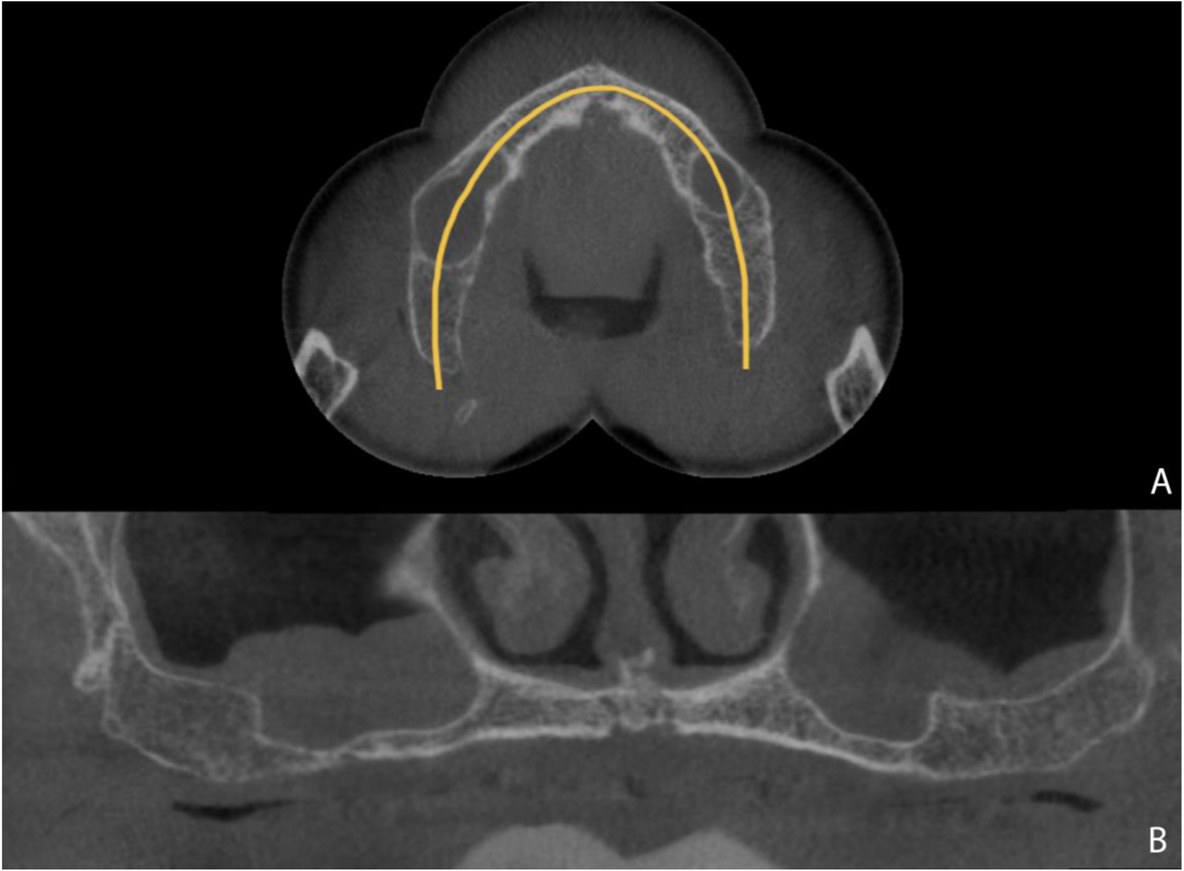
Maxillary computed tomography. **a** Axial view. **b** Coronal view

In the preoperative evaluation, all patients were clinically assessed for oral health status, the amount of keratinized gingiva, and the characteristics for rehabilitation: interocclusal space, inter-arch relationship (horizontal/vertical overlap), smile line, support lip, and vertical dimension. Patients with loss of size or occlusal instability were rehabilitated with a provisional total prosthesis before implant placement. All selected patients underwent maxillary computed tomography (CT) scan (Fig. 2) and intra- and extra-oral photographs were recorded. Surgical guides and bio-models were developed.

Individuals were asked to answer the OHIP-14 questionnaire. The instrument includes seven domains of the impact of life to be measured: functional limitation, physical pain, psychological discomfort, physical disability, psychological disability, social disability, and handicap. The answers given are according to a scale coded as 0 = never, 1 = rarely, 2 = sometimes, 3 = often, and 4 = always. The higher the value attributed by the respondent, the worse the perception of the problem [1].

### Surgical procedures

External antisepsis was performed on the face using 2% Riorex chlorhexidine digluconate (Rioquímica, São José do Rio Preto, SP, Brazil) and intrabuccal (intraoral) antisepsis was performed using digluconate 0.12% chlorhexidine (Colgate, São Paulo, SP, Brazil). The entire maxilla was anesthetized using 4% articaine and 1:100,000 epinephrine (DFL, Rio de Janeiro, Brazil). A side window of approximately 1.5 cm in diameter was made on the right and left maxilla using a long drill (diamond, spherical, 3018HL, KGSorensen, Cotia, SP, Brazil). The lifting of the sinus membrane was performed with the aid of curettes (Hu-Friedy, Chicago, USA).

**Fig. 3:**
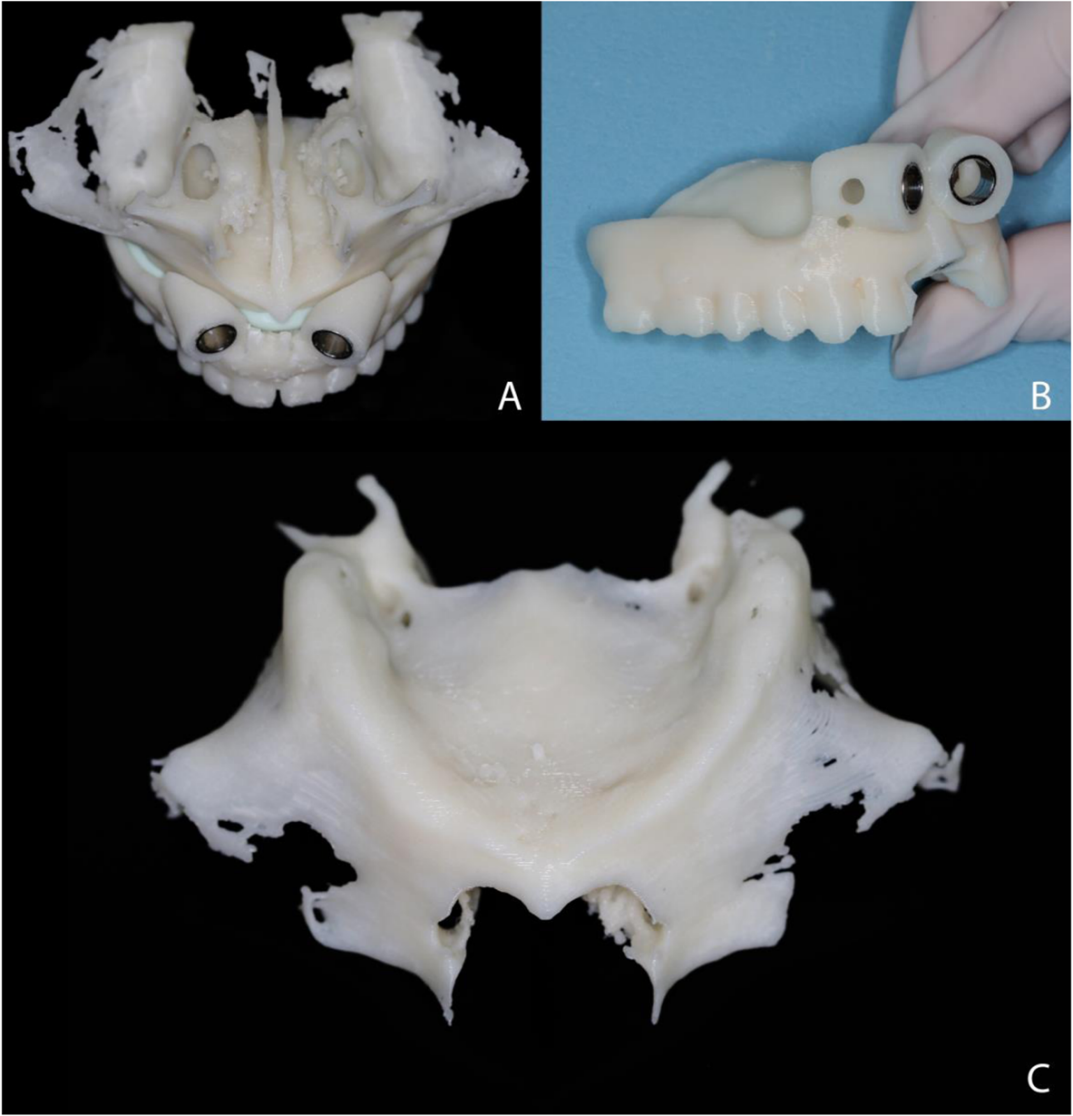
Surgical guide. **a** Surgical guide in occlusal view. **b** Surgical guide in frontal view. **c** Prototyped model of the atrophic maxilla

**Fig. 4:**
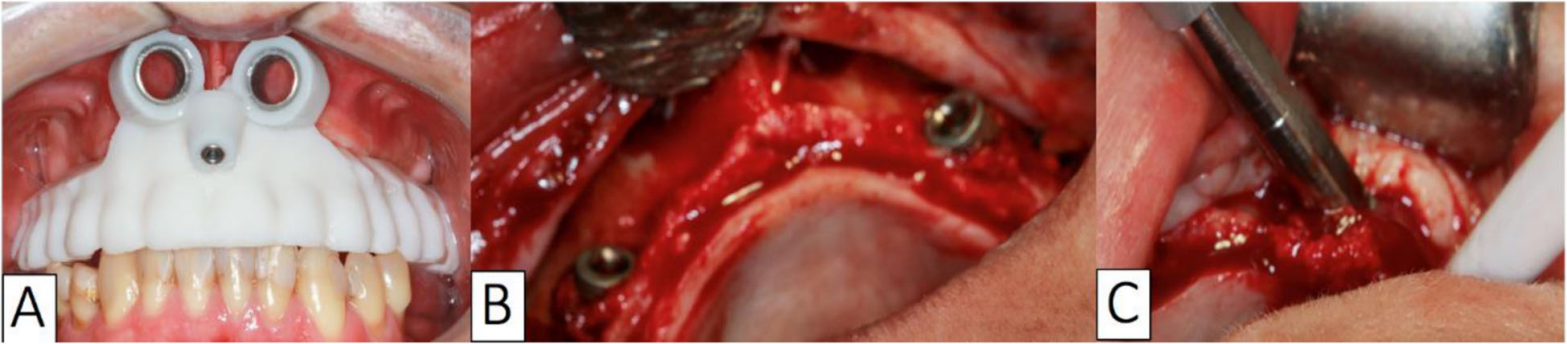
Insertion of the implant. **a** Prototyped surgical guide installed and stabilized. **b** Long transmaxillary implants are placed on both sides of the maxilla. **c** Long transmaxillary implant placement with the final insertion using a ratchet or tufted wrench

Following this, the prototyped surgical guide (Fig. 3) was installed and stabilized, and the guide was positioned for spear drilling (Fig. 4).

**Fig. 5.**
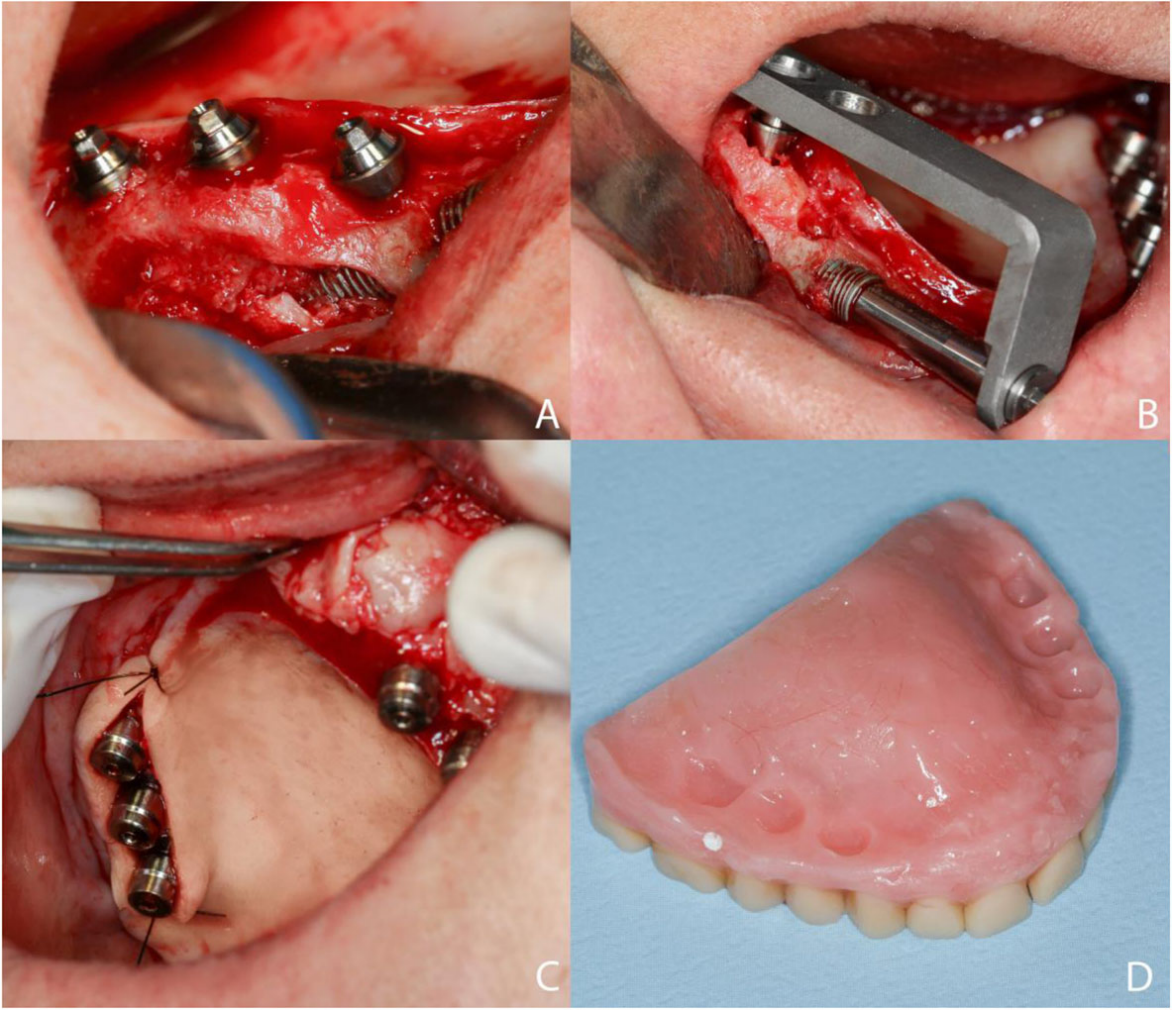
**a** Mini-pillar guide. **b** Installation of the mini-pillars. **c** Protectors on the mini-pillars. **d** Relief of the prosthesis on the mini-pillars

After initial drilling, guide and drilling using a long milling cutter (3.0 mm, spiral, lance type) were performed up to the length required for the implant placement. The implant was placed using a contra-angle mount (Fig. 4b), with the final insertion using a ratchet or tufted wrench (Fig. 4c). The mini-abutment guide was placed, coupled to the implant placed, and with the use of a guide set and more milling cutter to install the mini-abutments, to ensure drilling accuracy. Subsequently, the mini-pillars were placed (KOPP, Curitiba, PR, Brazil), according to the length of the transmucosal between the Morse cone and the gingival margin. Simple sutures were used to reposition the folded soft tissue (Fig. 5).

The prescription included 0.2% chlorhexidine digluconate mouthwash to be used three times a day, starting 2 days before the procedure until 10 days after; paracetamol 500 mg, every 6 h for 4 days; dexamethasone 4 mg, every 12 h for 2 days; and amoxicillin 875 mg, every 12 h for 7 days. In case of persistent pain, Tramadol 50 mg was prescribed.

### Prosthetic protocol

Six months after the placement of the long transmaxillary implants horizontally, the mini pillar protectors were removed, which were molded to make a plaster model with analogs to fabricate the prostheses in a specialized prosthetic laboratory. Transfers (Kopp Implant System, Curitiba, PR, Brazil) were used.

An acrylic base plate with a wax wheel for recording the positioning of the teeth and/or definition of the vertical dimension of occlusion was tested. After laboratory work, the set returned with the stock teeth to check the new dental relationship between the arches. Following this, the work proceeded to follow the conventional steps for the definitive fabrication of a protocol-type prosthesis (fixed acrylic), or another with a removable device (MK1®), in case recovery of the lip volume was required.

**Fig. 6:**
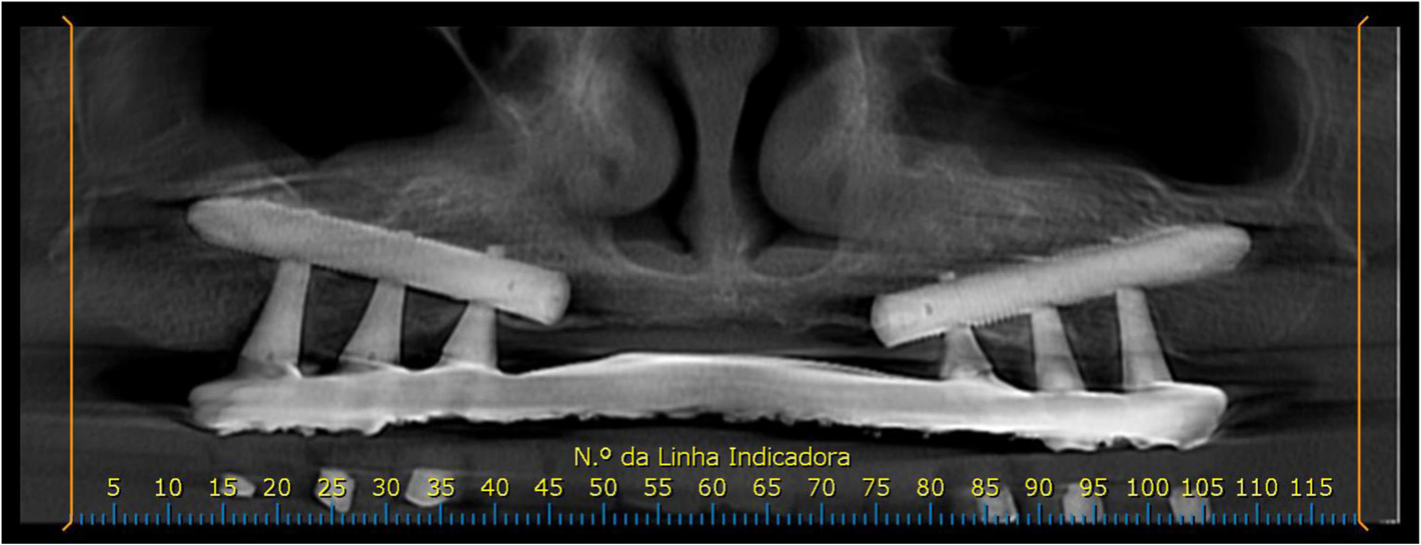
Tomographic scan of the follow-up

**Fig. 7:**
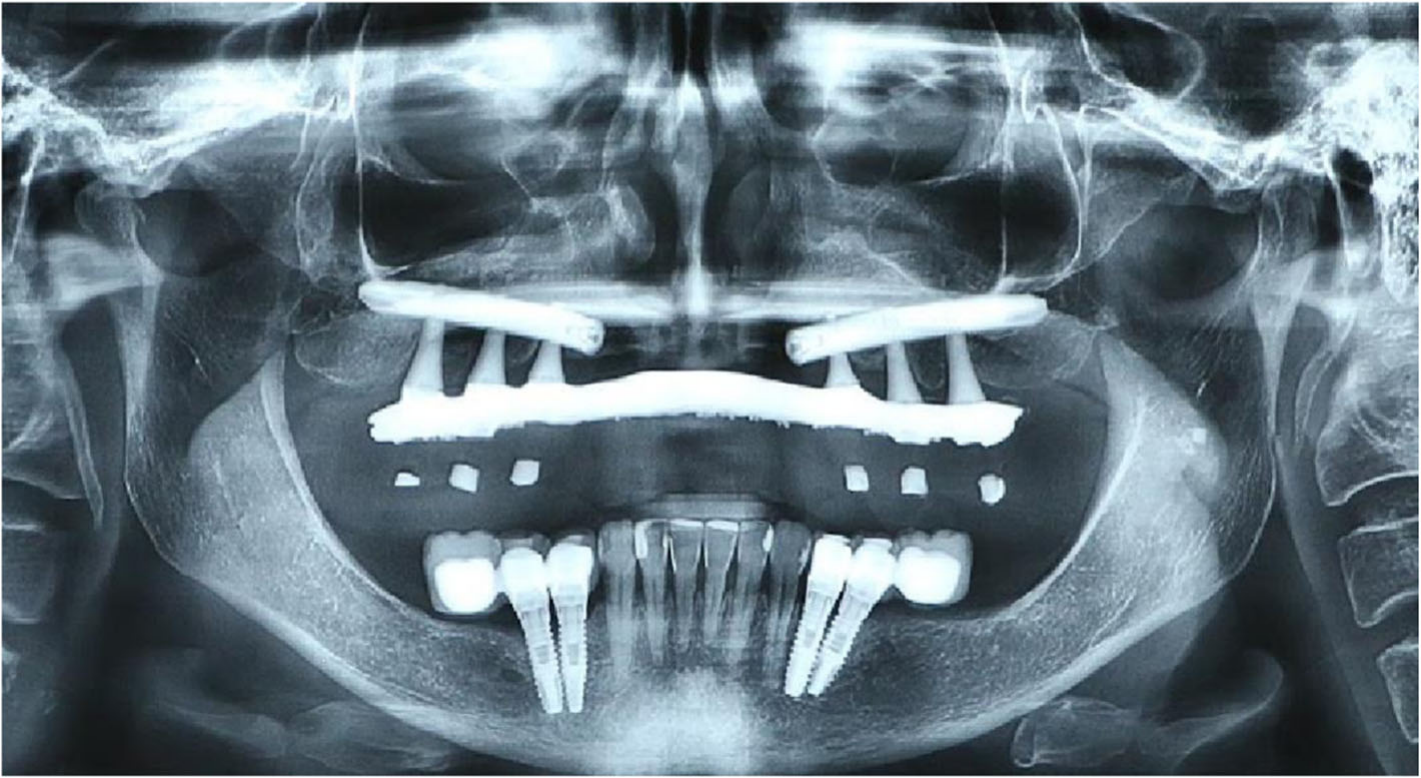
Panoramic radiography of the follow-up

**Fig. 8:**
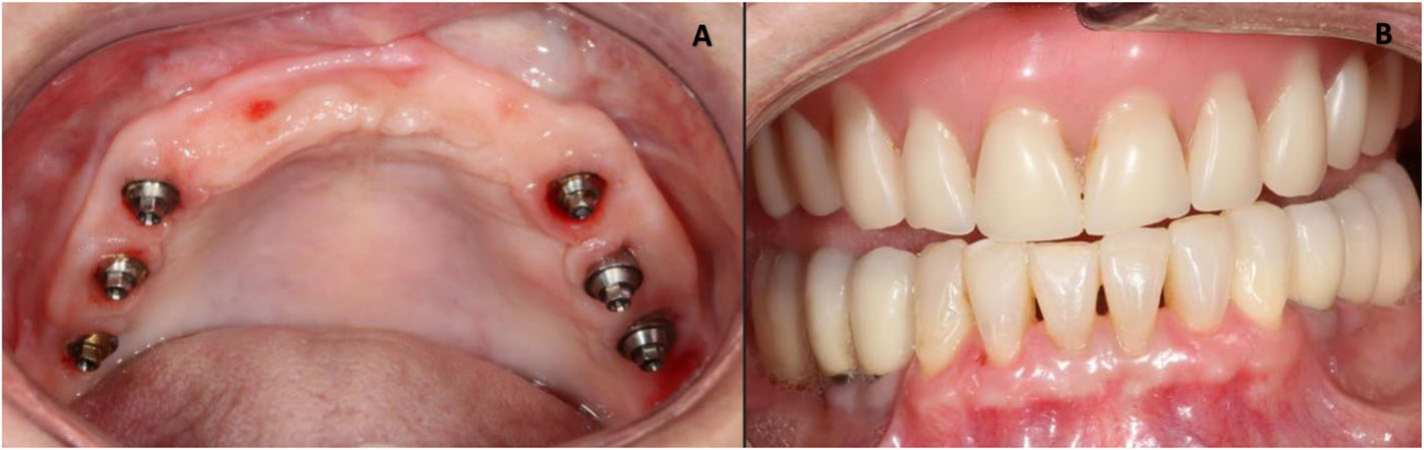
Final aspect

At the 6-month follow-up consultation after prosthetic rehabilitation, patients answered the OHIP-14 questionnaire again. A tomographic evaluation was performed (Figs. 6, 7, and 8).

### Statistical analysis

The results were statistically analyzed using descriptive and inferential analyses. The numerical variables of OHIP-14 were assessed using the Shapiro–Wilk normality test, which showed a non-normal distribution. Additionally, the reliability of this questionnaire was estimated using Cronbach’s alpha test, with a result of 0.78. To evaluate OHIP-14 with time, the Wilcoxon test of paired samples was used.

Values of *p* < 0.05 were considered statistically significant. The data were analyzed using the computer program IBM SPSS v.26.0 (Statistical Package for Social Science, Chicago, USA).

## Results

The study consisted of 12 individuals, with 10 (83.3%) females and two (16.7%) males. The mean age of the sample was 55.83 ± 2.78 years.

A total of 12 maxillary atrophic patients were rehabilitated, 10 using the transmaxillary implants. Of these, two patients rehabilitated with the transmaxillary implant required postoperative adjustments because the implants were lost. In the first case, it was necessary to remove the right-side implant due to infection during grafting. On the left side, the transmaxillary implant was kept in a horizontal position, and on the right side, the conventional implant was placed. In the second case, due to the indication of a graft, which would be performed on the same day of the implant placement, the patient opted for a conventional implant.

None of the patients registered with biological complications, such as perforation of the sinus membrane, mucositis, and peri-implantitis. All patients presented recovery of soft tissues, without infection of the maxillary sinus, paresthesia, fistula or abscess, or mechanical or prosthetic complications, such as a fracture of the implant or any prosthetic component.

It was possible to observe an improvement in the perception of OHRQoL between the pre- and postoperative periods in the OHIP-14 total score and the domains related to functional limitation (D1), physical pain (D2), psychological discomfort (D3), psychological disability (D5), social disability (D6), and handicap (D7) (*p* < 0.05; Table 1).

**Table 1:** Comparison of OHIP-14 and its domains in the pre-and postoperative periods

OHIP-14	Preoperative	Postoperative	***P*** value
	Median (min-max)		
D1	6 (2–8)	0 (0–3)	**0.002**
D2	5 (1–8)	0 (0–3)	**0.002**
D3	7.5 (1–8)	0 (0–4)	**0.002**
D4	7.5 (2–8)	0 (0–1)	**0.002**
D5	8 (3–8)	0 (0–4)	**0.002**
D6	2 (0–8)	0 (0–0)	**0.003**
D7	4 (8–8)	0 (0–0)	**0.003**
**Total**	38 (21–53)	1 (0–8)	**0.002**

Wilcoxon test of paired samples with a significance level of 0.05. Bold values indicate statistical significance. OHIP-14 domains: D1, functional limitation; D2, physical pain; D3, psychological discomfort; D4, physical disability; D5, psychological disability; D6, social disability; D7, handicap

## Discussion

Considering the particularities of rehabilitation treatments with implants, such as patients with the atrophic edentulous maxilla, and particularly rehabilitation using long implants, this study aimed to assess the impact of using a modified long transmaxillary implant, placed horizontally on the OHRQoL of patients with the atrophic maxilla.

The missing teeth impair mastication and swallowing. Additionally, lack of lip support affects esthetics and alters the individual’s speech; moreover, conventional prosthetic rehabilitation using removable prostheses provides feasible stability or lack of retention [5]. Therefore, dental implants are used in edentulous jaws to improve the retention and stability of complete dentures [14]. In addition, implant treatment is an effective method for dental rehabilitation from the perspective of patient-reported outcomes [10].

Patient expectations from implant treatment have also changed over the years, and esthetics plays an important role in defining rehabilitation treatment plans. Two of the many important factors to be considered are the intended implant site and the quantity and quality of bone [16]. Branemark introduced the zygoma implant as an effective method in the management of the atrophic edentulous maxilla [6]. It has been demonstrated that zygomatic implants are a very efficient option in the rehabilitation of atrophic edentulous maxilla [2]. However, as these implants are three to four times longer than conventional implants, they require greater dexterity and skill from the surgeon, as the trajectory of the implant must be locked in the middle or upper third of the face, especially in the body of the zygomatic bone, passing through the interior of the maxillary sinus. Taking into account the length and direction of the implant, precision is essential to minimize the resulting risks, such as deviations from the insertion angle [3, 9].

Long trasmaxillary implants placed horizontally in the maxilla are kept at the limits of the lower third of the face, without invading noble areas in the upper region, unlike the zygomatic implants placed vertically. Transmaxillary implants are anchored from the canine abutment to the maxillary tuberosity for stability. The innovation resulted from the need to advance regarding patient safety and in the other techniques of long implants that have been practiced in recent years. After the installation of the prostheses, the patients continued to be followed up, demonstrating that rehabilitation of the atrophic maxilla was achieved and the treatment was favorable with the viability of the procedure, as well as to the advances regarding the long zygomatic implants.

In this case series, it was possible to conclude that there was an improvement in the perception of OHRQoL between the pre- and postoperative periods in the total OHIP-14 score and in the domains related to functional limitation (D1), physical pain (D2), psychological discomfort (D3), physical disability (D4), psychological disability (D5), social disability (D6), and social disadvantage (D7). All submitted patients had self-perception and improved OHRQoL, which may positively impacts people’s lives, also in the psychological-social aspects.

More patients and longer follow-up periods are necessary for better data evaluation. As the implant model was new, we were unable to compare our results to other similar studies. Based on the results of this study, we concluded that the rehabilitation of severely atrophic jaws with long implants placed horizontally can present a viable and safe treatment alternative and that long-term transmaxillary implant rehabilitation improves OHRQoL. However, further studies with long-term follow-up should be performed.

## Conclusion

From the results obtained in this study, we can conclude that the long transmaxillary implant placed horizontally has a qualitative advantage in technical terms, compared to the zygomatic implant placed vertically or inclined, particularly regarding the risks of invasiveness and complications. The transmaxillary implant is a long implant that can be placed horizontally in the maxilla, remaining within the limits of the lower third of the face, without invading noble areas in the upper region.

Moreover, rehabilitation treatment using transmaxillary implants improved the OHRQoL of the patients evaluated in this study.

## Data Availability

The datasets used and/or analyzed during the current study are available from the corresponding author upon reasonable request.
